# Risk Factors Associated with the Development of Tuberculosis Among HIV-Infected Patients in Khartoum in 2010

**DOI:** 10.3934/publichealth.2015.4.784

**Published:** 2015-12-02

**Authors:** Heitham Awadalla, Fateh El-Samani, Mohammed A. Soghaier, Mahgoub Makki

**Affiliations:** 1 Public Health Specialist, Federal Ministry of Health, Public Health Institute, Khartoum, Sudan; 2 Professor of Public Health, Ahfad University for Women, Omdurman, Sudan and WHO regional consultant; 3 Epdemiologist, Federal Ministry of Health, Directorate of Epidemiology and Zoonotic diseases, Khartoum, Sudan; 4 Community Physician, Federal Ministry of Health, National Medical Commission, Khartoum, Sudan

**Keywords:** HIV/AIDS, tuberculosis, voluntary counselling and testing, Sudan Federal Ministry of Health and National AIDS Control Program, TB-HIV

## Abstract

**Background:**

Tuberculosis (TB) screening among patients infected with Human Immunodeficiency Virus (HIV) is one of the approaches for controlling TB-HIV co-infection. The absence of typical TB symptoms among HIV-infected patients makes diagnosis challenging. Identifying predisposing risk factors of TB among HIV-infected patients could possibly guide TB diagnosis and treatment. This study was designed to identify some important factors associated with TB among HIV-infected patients and to quantify the strength of this association.

**Methodology:**

In 2010, a case control study was conducted in Khartoum State, Sudan. Cases and controls were selected by simple random sampling with a 1:2 ratio; 97 cases and 194 controls were enrolled in the study. A logistic regression model was built to estimate and quantify the strength of the association between the study variables and the outcome; a *p*-value less than 0.05 was considered the cut-off point for a significant statistical association.

**Results:**

Past history of TB, CD4 count < 200 cells/µl, late clinical stages, non-employment, and no formal education were found to be risk factors for developing TB among HIV-infected patients. The adjusted ORs and 95% CIs were (6.9: 3.75–12.99), (4.8: 1.57–15.26), (5.8: 1.88-17.96), (2.5: 1.26–5.03), and (2.5: 1.28–4.63), respectively. Poor adherence, marital status, age, and gender are not associated with developing TB among HIV patients.

**Conclusion:**

HIV patients who have at least one of the risk factors found in this analysis are at higher risk of TB; therefore, they should be screened more frequently and treated promptly, especially HIV patients with previous TB.

## 1. Introduction

Tuberculosis (TB) and Human Immunodeficiency Virus (HIV) pose significant global public health problems. They are overlapping epidemics [1]. Both have been declared global emergencies demanding global attention. Triggered by HIV infection, TB has emerged as a major public health threat in Sub-Saharan Africa [2]. HIV infection is a major risk factor for reactivation of latent TB and rapid progression of recent TB infection. On the other hand, TB increases HIV replication and is now a major cause of death among HIV-infected patients in developed and developing countries [3]. TB and HIV interaction has resulted in a parallel pandemic of TB disease, increasing the annual TB disease rates in sub-Saharan African from 5-fold to 10-fold during the latter half of the 1990s [4]. According to the Provider Initiated Testing and Counselling (PITC) Report 2009, in Sudan the prevalence of HIV among TB patients in TB clinical settings is estimated to be 18% [5].

In order to ensure effective collaboration between Acquired Immunodeficiency Syndrome (AIDS) and TB control programs, it is important to have a screening strategy to detect HIV among TB patients and a screening program to detect TB among HIV-positive patients. The former is easy and could be easily and quickly done through a blood test. The latter is still challenging in many countries because HIV-infected TB patients often lack the classic clinical symptoms of TB [6],[7]. Therefore, many studies had been conducted to determine the risk factors associated with TB among HIV-infected patients [8],[9],[10]. These factors include different socio-demographic factors, CD4 count, WHO-clinical stage, co-morbid conditions, and TB-presenting symptoms.

Moreover, Isoniazid Preventive Therapy (IPT) is one of the strategies that is used to reduce the burden of TB among HIV-infected patients [11],[12],[13]. However, IPT has not yet been implemented in Sudan because it requires a strong and efficient TB screening program among HIV-infected patients. Therefore, this study could possibly provide useful recommendations and guiding clinical algorithms for TB screening among HIV patients in Sudan.

## 2. Materials and methods

This multi-centered case control study was conducted in Khartoum State in Sudan between January and October 2010. There are six Voluntary Counselling and Testing/Anti-Retroviral Treatment VCT/ART centers in the cities of Khartoum, Omdurman, and Khartoum North 6 (21% of the total ART in the country). In these centers, 2,049 patients undergo Highly Active Antiretroviral Therapy (HAART). All of these centers provide screening services and treatment for TB and HIV-infected patients, free of charge. All six of these centers were included in the current study.

The cases (patients with TB-HIV co-infection) and the controls (only HIV-infected patients) were selected from these six VCT/ART centers using simple random sampling (SRS) after listing all of the patients that were receiving care. Selection of the cases was based on the World Health Organization (WHO) standard case definition. Inclusion criteria were: males and females with either pulmonary TB (PTB) or extra-pulmonary TB (EXPTB), and incident or prevalent TB. Patients under 15 years of age and those that did not reside in Khartoum State were excluded from the study.

Sample size was calculated assuming 95% confidence level (CI) and 80% power. Two controls were selected for each case to increase the study's power [14]. After screening for inclusion and exclusion criteria, a total of 97 cases and 194 controls were recruited for the study. Subjects that were initially selected as controls and then discovered to have TB or that developed TB later during the study, were reclassified as cases for the analysis and other controls were selected instead.

Data was collected by using structured questionnaires, reviewing patient registries, and screening results records. Six qualified counsellors, working within the six VCT/ART centers, were trained to collect data from the selected cases and controls in the same way using the structured questionnaire.

A multivariate logistic regression model with 95% CI was run through SPSS version 20 software to calculate the odds ratio (OR), thereby quantifying the associations between the occurrence of TB and the different predictors or risk factors [15]. The significance level was considered to be a *p*-value < 0.05.

### 2.1. Ethical considerations

Ethical approval was obtained from the Sudan Medical Specialization Board on 15^th^ February 2010. Written informed consent was obtained from both the cases and controls before enrolment in the study.

## 3. Results

A total of 291 subjects were enrolled in this study; 97 (33%) were cases (TB-HIV co-infection) and 194 (67%) were controls (HIV-infected only). The age of the cases and controls enrolled in this study was almost identical. However, the mean age was slightly higher among the controls [37.2 ± 9.4] compared to the cases [35 ± 9.8]. Table 1 summarizes the baseline characteristics of the cases and the controls.

In terms of the risk factors associated with the outcome, five variables were found to be significant for developing in HIV-infected patients. Past medical history of TB (HIV-infected participants with TB that was successfully treated before the current study) was found to be associated with developing TB (adjusted OR = 6.98 and 95% CI [3.75–12.99]). HIV patients with no formal education (i.e., the official primary, secondary, and higher education levels within the country's educational system) were found to have a higher risk of developing TB compared to HIV patients who were educated (adjusted OR = 2.54 and 95% CI [1.28–4.63]). Unemployed HIV patients were found to have a greater risk of developing TB (adjusted OR = 2.53 and 95% CI [1.26–5.03]). Patients with late clinical stage HIV based on WHO criteria or with a low CD4 count (less than 200 cells/µl) were found to be at a higher risk of developing TB in comparison to patients in early clinical stages of HIV (OR = 5.86 and 95% CI (1.88–17.96) and OR = 4.81 and 95% CI (1.57–15.26), respectively). Other sociodemographic and clinical factors—gender, age, and marital status—did not show a significant statistical association with the study outcome. Table 2 shows the final multivariate logistic regression model estimating the association between the factors and the study outcome.

**Table 1.** The baseline characteristics of the study participants.

Characteristics	Cases (TB-HIV) n (%)	Controls (HIV patients) n (%)
**No. of participants**	97 (33%)	194 (67%)
**Age in years (mean ±SD)**	35.8 ± 9.8	37.2 ± 9.4
**Male sex**	64 (66%)	102 (53%)
**Marital status**		
Single	29 (30%)	41 (21%)
Married	43 (44%)	94 (48%)
Divorced	18 (19%)	31 (16%)
Widowed	7 (7%)	28 (15%)
**Employment**		
Officer (professional)	2 (2%)	27 (14%)
Labor (skilled & non-skilled )	43 (44%)	84 (44%)
Student	1(1%)	6 (3%)
Unemployed	51(53%)	77 (39%)
**Education**		
No formal education	37(38%)	32(17%)
Primary	33(34%)	59(30%)
Intermediate	9(9%)	52(27%)
Higher	18(19%)	51(26%)
**Other co-morbid condition**	4 (4%)	10 (5%)
**Past history of TB**	66 (68%)	39 (20%)
**Baseline CD4 count cells/µl (median: IQR)**	112(82-156)	132(92-200)
**WHO clinical stage**		
Stage 1	1 (1%)	28 (15%)
Stage 2	2 (2%)	21 (11%)
Stage 3	81 (84%)	130 (69%)
Stage 4	12 (13%)	10 (5%)
All patients receiving HIV care (mean± SD)	16.3±13.4	21.2±15.6
Patients receiving HAART (mean± SD)	20.9±13.9	28.5±25.5
**HIV patients on HAART n (%)**	62 (64%)	142 (74%)
**Duration of HAART in months (mean± SD)**	17.2±13.8	22.1±15.0
**Period before start of HAART (in months)**	3.6±5.5	6.3±22.6
**Adherence to HAART**		
Good	49 (79%)	111 (78%)
Fair	9 (15%)	26 (18%)
Poor	4 (7%)	5 (4%)

**Table 2.** Multivariate logistic regression model and adjusted OR for the possible risk of developing TB among HIV-infected patients.

Characteristics	Adjusted OR	95%CI	*p*-value
**Male sex**	1.99	0.92-4.32	0.07
**Age**	0.99	0.95-1.02	0.45
**Past TB history**	6.98	3.75-12.99	< 0.01
**No formal education**	2.54	1.28-4.63	< 0.01
**Married (not single)**	2.20	0.79-4.38	0.07
**Unemployed**	2.53	1.26-5.03	0.01
**Late clinical WHO stages**	5.86	1.88-17.96	< 0.01
**CD4 less than 200 cells/µl**	4.81	1.57-15.26	0.01
**Poor adherence**	1.81	0.47-7.04	0.39
**Unemployed**	9.52	2.17-41.74	< 0.01

## 4. Discussion

HIV-associated immunodeficiency is an important risk factor for the recurrence of TB, whether that disease is caused by relapse or exogenous re-infection [16]. Like many other studies, patients with a past history of TB had a higher chance of developing it if they were also infected with HIV [15],[17]. Evidence suggests that the restoration of Mycobacterium tuberculosis-specific immunity is incomplete during the first year (at least) that an HIV-infected patient undergoes HAART [18],[19]; therefore, it is certainly plausible that a previous history of TB would persist as a significant risk factor for incident TB after the initiation of HAART. Some studies found that the association between the risk of TB and a past history of TB was weaker than the association observed in this current study. Most of the cases in the previous studies that reported a weak association had completed their anti-TB drugs within one year or less before enrollment in HAART. Therefore, the short time duration after completing anti-TB medications reduces the probability of re-infection in these cases [20].

Based on the WHO clinical stages for HIV, stage 3 and stage 4 were found to be strong predictors of TB among HIV-infected patients. This result is consistent with current available clinical knowledge and it has been confirmed by several similar studies [21]–[25]. The possibility of a missed-diagnosis of TB among stage 4 HIV patients is likely to occur because these patients usually lack the typical signs and symptoms associated with TB. Therefore, it is possible that an even stronger association exists between stage 4 HIV and TB risk than what we found our study.

A low CD4 cell count of less than 200cell/µl remains an independent predictor for TB among HIV-infected patients [26],[27]. The association between the risk of TB and low CD4 count in this present study (*p*-value 0.03) was not as strong as we had expected. This could be due to several factors; either most of control patients had just completed their TB treatment or they had TB while they were undergoing long-term HIV treatment HIV before enrolling in the study. This explanation can be supported by two results obtained from the study. First, the mean duration of HIV was much higher among the controls (21 months) than the cases (16 months) with a *p*-value of 0.01. Second, 20% of the controls had a past history of TB, and the possibility of contracting TB while also living with a long-term HIV infection was highly probable.

Education level and occupation showed a significant association with TB risk among the HIV-infected patients in this study. Poor educational level and no employment were both associated with higher risk of majority of infectious diseases. Occupation was most likely determined by the level of education [28]. Most of the non-skilled labors and those without any occupation probably had a low level of education. Therefore, both poor educational level and no employment acted synergistically to increase TB risk.

In some studies, a longer duration of HAART was found to be associated with a lower TB risk [12],[29]. Pre-treatment immunodeficiency (gap before starting HAART) and the lack of response to HAART are considered to be principal risk factors for TB [30]. However, this association was not found in this study. This could be explained by the fact that most of the HIV patients enrolled in this study were either in clinical stage 3 or stage 4 and they were eligible for HAART treatment (almost matched for this variable).

Poor adherence to treatment has the same effect as not taking the treatment. The measurement of adherence was challenging. Moreover, it is typically subjected to many other confounders that were not addressed in this study, such as the medical condition of the patient, whether or not the patient is taking other types of medications, and the stage of the patient's disease (late stages versus early stages). To overcome these challenges, we recommend a follow up cohort study design instead of a retrospective design [31].

In univariate logistic regression analysis of this study, an association was found between male sex and developing TB among HIV-infected patients. A similar result was obtained from similar studies conducted in developing countries, such as Tanzania [10], Thailand [32], and South Africa [31]. However, multivariate analysis and the findings presented in other studies revealed that sex is not a risk factor [33]–[35]. This could be explained by the effect of some additional confounders, such as the CD4 level and the WHO-clinical stage.

## 5. Conclusion

In conclusion, TB risk was found to be higher among unemployed participants and those with a low educational level. The most important and absolute predictors for TB risk among HIV-infected patients were: a low baseline CD4 count of less than 200cell/µl, late WHO clinical stages, and past history of TB. Nevertheless, TB clinical screening should be conducted regularly for HIV patients whether or not these predictors are present. HIV patients who are expected to be at a higher risk for TB should be examined and investigated thoroughly and more frequently. Evidence of the role played by an earlier start and good adherence to HAART was not strong enough in this study; however, the recommendation for an earlier start of HAART and good adherence remains valid.

## Abbreviations

AIDS: Acquired Immunodeficiency Syndrome
ART: Anti-Retroviral Treatment
TB: Tuberculosis
HIV: Human Immunodeficiency Virus
WHO: World health Organization
SRS: Simple Random Sampling
HAART: Highly Active Antiretroviral Therapy
VCT: Voluntary Counselling and Testing
SNAP: Sudan National AIDS Control Program
PITC: Provider Initiated Testing and Counselling
IPT: Isoniazid Preventive Therapy
PTB: Pulmonary TB
EPTB: Extra Pulmonary TB

## References

[1] Dean AS, Zignol M, Falzon D (2014). HIV and multidrug-resistant tuberculosis: overlapping epidemics. Eur Resp J.

[2] Zumla A, Petersen E, Nyirenda T (2015). Tackling the tuberculosis epidemic in sub-Saharan Africa–unique opportunities arising from the second European Developing Countries Clinical Trials Partnership (EDCTP) programme 2015-2024. Int J Infect Dis.

[3] Adeiza MA, Abba AA, Okpapi JU HIV-Associated tuberculosis (2014). A sub-saharan african perspective. Sub-Sah Afr J Med.

[4] Zumla A, George A, Sharma V (2015). The WHO 2014 Global tuberculosis report—further to go. The Lancet Global Health.

[5] Federal Ministry of Health-Sudan (2009). Provider Initiated Testing and Counselling (PITC) Report. Sudan National AIDS Control Program (SNAP).

[6] Alemayehu M, Gelaw B, Abate E (2014). Active tuberculosis case finding and detection of drug resistance among HIV-infected patients: A cross-sectional study in a TB endemic area, Gondar, Northwest Ethiopia. Intl J Mycobacter.

[7] Kisembo H, Den Boon S, Davis J (2014). Chest radiographic findings of pulmonary tuberculosis in severely immunocompromised patients with the human immunodeficiency virus. Br JRadiol.

[8] Getahun H, Gunneberg C, Granich R (2010). HIV infection—associated tuberculosis: The epidemiology and the response. Clinl Infect Dis.

[9] Moore D, Liechty C, Ekwaru P (2007). Prevalence, incidence and mortality associated with tuberculosis in HIV-infected patients initiating antiretroviral therapy in rural Uganda. Aids.

[10] Liu E, Makubi A, Drain P (2015). Tuberculosis incidence rate and risk factors among HIV-infected adults with access to antiretroviral therapy. AIDS (London, England).

[11] Bucher HC, Griffith LE, Guyatt GH (1999). Isoniazid prophylaxis for tuberculosis in HIV infection: a meta-analysis of randomized controlled trials. Aids.

[12] Kufa T, Mabuto T, Muchiri E (2014). Incidence of HIV-associated tuberculosis among individuals taking combination antiretroviral therapy: a systematic review and meta-analysis.

[13] Dowdy DW, Golub JE, Saraceni V (2014). Impact of isoniazid preventive therapy for HIV-infected adults in Rio de Janeiro, Brazil: an epidemiological model. J Acqui Immune Defic Syndr (1999).

[14] Wacholder S, Silverman DT, McLaughlin JK (1992). Selection of controls in case-control studies: III. Design options. Am J Epidemiol.

[15] Lawn S, Harries A, Williams B (2011). Antiretroviral therapy and the control of HIV-associated tuberculosis. Will ART do it?. IntJ TubercLung Dis.

[16] Korenromp E, Scano F, Williams B (2003). Effects of human immunodeficiency virus infection on recurrence of tuberculosis after rifampin-based treatment: an analytical review. Clin Infect Dis.

[17] Kumar A, Kumar A, Gupta D (2012). Global guidelines for treatment of tuberculosis among persons living with HIV: unresolved issues [Perspectives]. Inter J Tuberc Lung Dis.

[18] Lawn S, Bekker L, Wood R (2005). How effectively does HAART restore immune response to Mycobacterium Tuberculosis? Implication for tuberculosis control. AIDS Journal.

[19] Hsu DC, Kerr SJ, Thongpaeng P (2014). Incomplete restoration of Mycobacterium tuberculosis-specific-CD4 T cell responses despite antiretroviral therapy. J Infect.

[20] Badri M, Wilson D, Wood R (2002). Effect of highly active antiretroviral therapy on incidence of tuberculosis in South Africa: A cohort study. Lancet.

[21] Elliot AM, Luo N, Tembo G (1990). Impact of HIV on tuberculosis in Zambia: a cross-sectional study. BMJ.

[22] WHO, CDC, IUALTD (2002). Community TB care in Africa. Report on a ‘Lesson Learned’ meeting in Harare, Zimbabwe, 27-29 September 2000.

[23] Alene KA, Nega A, Taye BW (2013). Incidence and predictors of tuberculosis among adult people living with human immunodeficiency virus at the University of Gondar Referral Hospital, Northwest Ethiopia. BMC Infect Dis.

[24] Kassa A, Teka A, Shewaamare A (2012). Incidence of tuberculosis and early mortality in a large cohort of HIV infected patients receiving antiretroviral therapy in a tertiary hospital in Addis Ababa, Ethiopia. Trans R Soc Trop Med Hyg.

[25] Ciaranello A, Lu Z, Ayaya S (2014). Incidence of World Health Organization stage 3 and 4 events, tuberculosis and mortality in untreated, HIV-infected children enrolling in care before 1 year of age: an IeDEA (International Epidemiologic Databases To Evaluate AIDS) east Africa regional analysis. Pediatr Infect Dis J.

[26] Middelkoop K (2011). The effect of HIV and an Antiretroviral treatment programme on Tuberculosis transmission, incidence and prevalence in a South African Township.

[27] Pupaibool J, Limper AH (2013). Other HIV-associated pneumonias. Clin Chest Med.

[28] Cagney KA, Lauderdale DS (2002). Education, wealth, and cognitive function in later life. The Journals of Gerontology Series B: Psychological Sciences and Social Sciences.

[29] Iroezindu M, Ofondu E, Hausler H (2013). Prevalence and risk factors for opportunistic infections in HIV patients receiving antiretroviral therapy in a resource-limited setting in Nigeria. J AIDS Clinic Res S.

[30] Sinha S, Shekhar RC, Singh G (2012). Early versus delayed initiation of antiretroviral therapy for Indian HIV-Infected individuals with tuberculosis on antituberculosis treatment. BMC Infect Dis.

[31] Stephen Da l, Motasim B, Robina W (2005). Tuberculosis among HIV-infected patients receiving HAART: long term incidence and risk factors in a South African cohort. AIDS.

[32] Putong N, Pitisuttithum P, Supanaranond W (2002). Mycobacterium tuberculosis infection among HIV/AIDS patients in Thailand: clinical manifestations and outcomes. Southeast Asian J Trop Med Public Health.

[33] Sudre P, Hirshel B, Toscani L (1996). Risk factors for tuberculosis among HIV-infected patients in Switzerland. Eur Respir J.

[34] Brussard P, Remis RS (1999). Incidence of tuberculosis among reported AIDS cases in Quebec from 1979 to 1996. JAMC.

[35] Verma S, Dhungana G, Joshi H (2012). Prevalence of pulmonary tuberculosis among HIV infected persons in Pokhara, Nepal. J Nepal Health Research Council.

